# Application of Liquid Chromatography Coupled to Mass Spectrometry in Quality Assessment of Dietary Supplements—A Case Study of Tryptophan Supplements: Release Assay, Targeted and Untargeted Studies

**DOI:** 10.3390/ph15040448

**Published:** 2022-04-04

**Authors:** Krzysztof Adam Stępień, Joanna Giebułtowicz

**Affiliations:** Department of Bioanalysis and Drugs Analysis, Faculty of Pharmacy, Medical University of Warsaw, 1 Banacha, 02-097 Warsaw, Poland; krzysztof.stepien@wum.edu.pl

**Keywords:** dietary supplement, food supplement analysis, LC-MS/MS, release test, quality control, food composition

## Abstract

Dietary supplements are widely consumed in the EU and the USA. Based on their similarity to pharmaceuticals, consumers mistakenly believe that dietary supplements have also been approved for safety and efficacy. However, in the absence of mandatory testing, data on supplement quality is scarce. Thus, we applied liquid chromatography coupled with tandem mass spectrometry to analyse the quality of dietary supplements containing tryptophan (Trp). We examined 22 supplements in tablets or capsules, produced in the USA, Great Britain, Germany, France, Czech Republic, and Poland. Trp release, crucial for bioavailability and efficiency, was assessed. Additionally, we performed a qualitative analysis of the main ingredient and screened for contaminants. Among the contaminants, we detected Trp’s metabolites, condensation products of Trp and carbonyl compounds, Trp degradation products, degradation products of kynurenine, and other contaminants such as glucosamine and melatonin. The main ingredient content was in the range of 55–100% in capsules and 69–87% in tablets. Surprisingly, almost no Trp release was noted from some supplements. Our study confirms the need to advance research on supplements. We believe that the high-quality analysis of supplements based on reliable analytical techniques will be an important contribution to the discussion on the regulatory framework of these products.

## 1. Introduction

The consumption of dietary supplements is increasing globally, due to low prices, broad prescription-free distribution, and a common belief in efficacy and safety [1]. In the EU, they are classified as food and contain ingredients with nutritional or physiological effects [2]. Similarly, in the USA, dietary supplements are also classified as food and contain amino acids, herbal substances, vitamins, minerals, and enzymes [3]. Although they are foods, they are sold in typically pharmaceutical dosage forms such as tablets, capsules, sachets, and others designed to be taken in small and defined unit quantities. Based on their similarity to pharmaceuticals, consumers mistakenly believe that dietary supplements have also been approved for safety and efficacy before marketing. However, in the absence of mandatory quality testing, data on supplement quality is scarce. When the data do appear, they indicate some issues such as the presence of contaminants [4,5], the content of the main ingredient lower than the declared one [6,7], or low release from the formulation [8]. The most commonly described contaminants are heavy metals [9], anabolic steroids in preparations for athletes [10], and dioxins in dietary supplements containing fish oil [11]. The amount of the main ingredient in the dietary supplements was only examined for melatonin supplements [1] and supplements containing eicosapentaenoic acid (EPA) and docosahexaenoic acid (DHA) [12]. Release of the main constituent from the formulation was conducted for formulations with calcium carbonate [13], melatonin [14], folic acid [15,16], iron [17], triiodothyronine [18], trans-resveratrol [19] and lutein [8]. The paucity of data on supplement quality is disadvantageous [20]. Therefore, the need for international collaboration to advance the knowledge on supplements has recently been emphasized. Not only the efficacy but also the quality of dietary supplements should be evaluated, which is essential to improve the regulatory framework [21].

Depression, the most common mental illness, is one of the most common disorders for which supplementation is also used. This disorder affects over 300 million people around the world, of different ages, and in all communities [22]. The prevalence of the illness increases with age and is more common in women and people with higher education [23]. Depression is treated pharmacologically, often with moderate efficacy [24]. This is why some people also use supplementation. There are numerous mood-enhancing supplements on the market, mainly containing the neutral amino acid tryptophan (Trp) [25]. It is one of the 20 L-amino acids incorporated into proteins during mRNA translation [26] and a precursor of serotonin (5-hydroxytryptamine), niacin (niacinamide), and melatonin. Trp enters the kynurenine pathway and is a precursor of the coenzyme NAD(P)+ [27]. It is an exogenous amino acid whose [28] deficiency leads to the insufficient synthesis of the neurotransmitter serotonin, which worsens mood. Lower levels of Trp in peripheral blood have been confirmed in patients suffering from depression. Trp supplementation significantly improved the symptoms of the disease [29]. Trp has a positive effect on mood, cognitive functions [30], sleep [31], and a decrease or maintenance of a healthy weight [32]. Response to supplementation is individual and may be influenced by genetic factors [33]. The potential efficacy of Trp in depression patients led us to select supplements with this ingredient to evaluate their quality.

Western diet usually contains about 0.5 g of Trp per day. However, only 2–3% of this amount enters the brain for conversion, via 5-hydroxytryptophan, to serotonin. It is due to extensive metabolism and competition with other long-chain neutral amino acids, e.g., histidine, isoleucine, leucine, methionine, phenylalanine [34]. Trp is an ingredient of dietary supplements [35]. Sometimes, during Trp supplementation, dose-independent side effects occur, i.e., tremors, dry mouth, mild nausea, dizziness. Contaminants, present in commercially available Trp for nutritional use (feed-grade Trp in raw materials of different manufacturers), were investigated [36]. The contaminants detected and identified in commercially available Trp sources were the known metabolites of this amino acid, oxidation products of Trp, condensation products of Trp with carbonyl compounds [37]. So far, there are little data on the quality of Trp preparations. Additionally, no data have been published on the content and release of Trp from supplements, and most studies on supplements rely on simple analytical techniques.

This study aimed to apply liquid chromatography coupled with mass spectrometry as a highly reliable analytical technique to evaluate the quality of dietary supplements containing Trp in tablets or capsules (*n* = 22) produced in the USA, UK, Germany, France, Czech Republic, and Poland. This evaluation was performed by (i) assessment of Trp release, a key parameter for bioavailability and efficacy, (ii) qualitative analysis of the main ingredient, and (iii) screening for contaminants. We believe that a high-quality analysis of supplements will be an important contribution to the discussion of the regulatory framework for these products and that the new analytical approach will have broad applicability in the assessment of supplement quality.

## 2. Results and Discussion

### 2.1. Tentative Contaminants Present in Trp Supplements

**Figure A1:**
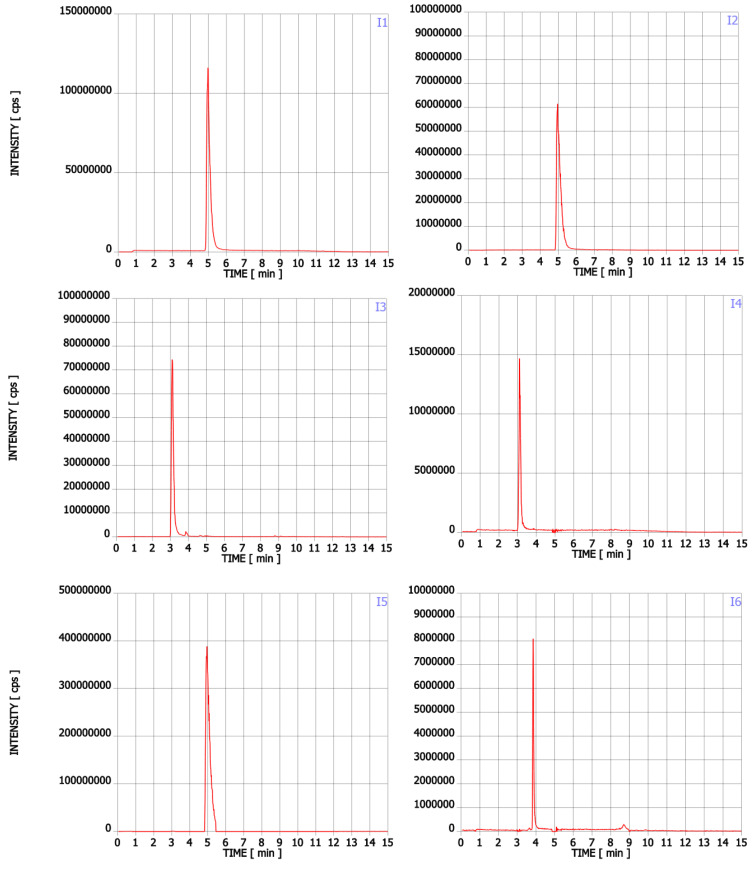

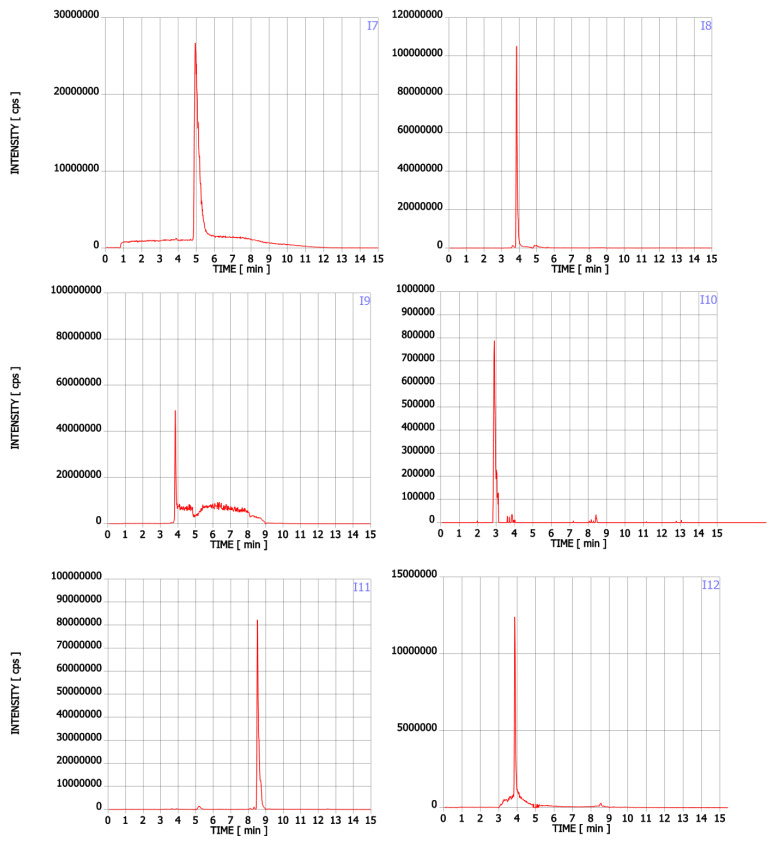

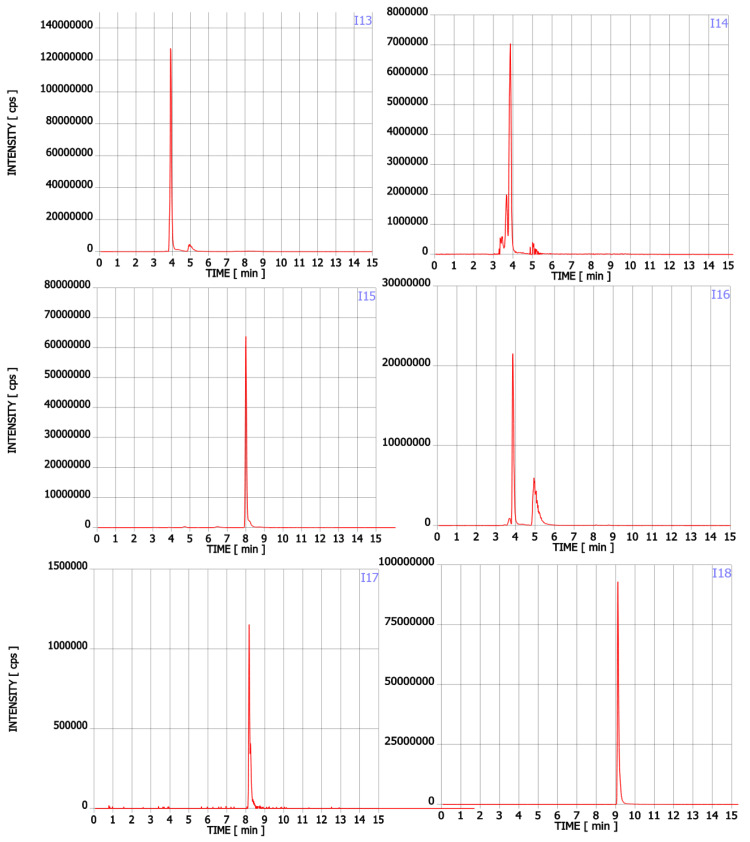

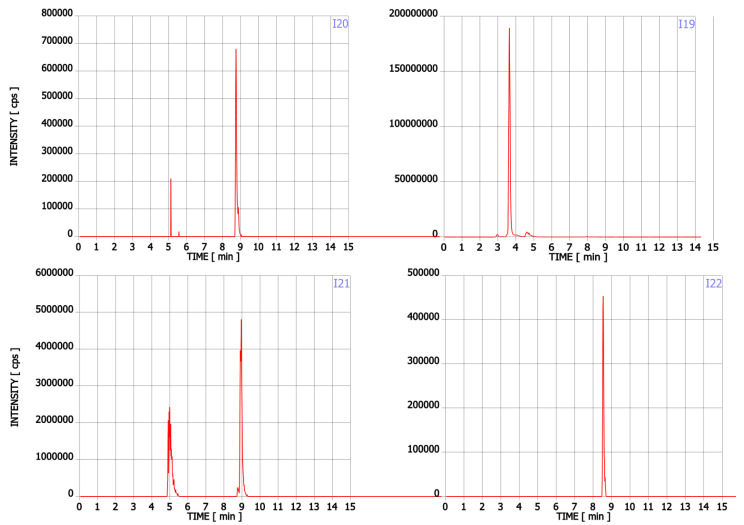
Chromatograms of Trp contaminants (I1-I22) detected in Trp dietary supplements.

In addition to Trp, twenty-two compounds were detected in the analysed supplements in the range of 0.02% to 43.89% of the main ingredient area (Table 1, Figure A1). Their molecular formula, retention time, experimental and theoretical mass, fragmentation, and tentative names are presented in Table 1. None of these compounds was listed on the package as a component of the supplement. Detected compounds were classified into five groups: (A) Trp’s metabolites, (B) condensation products of Trp and carbonyl compounds, (C) Trp degradation products, (D) degradation products of kynurenine and (E) other contaminants.

**Figure 1:**
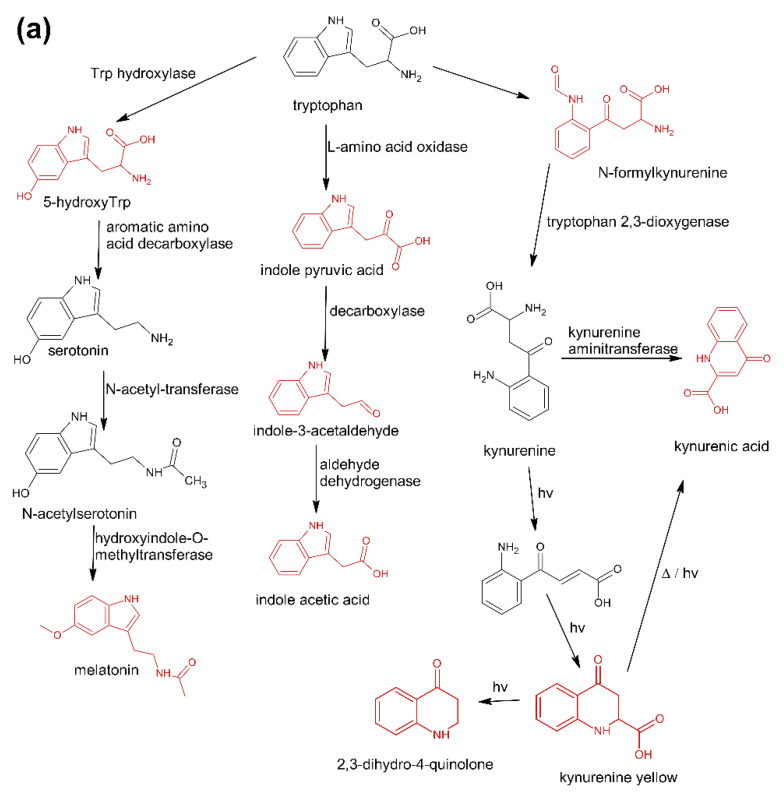

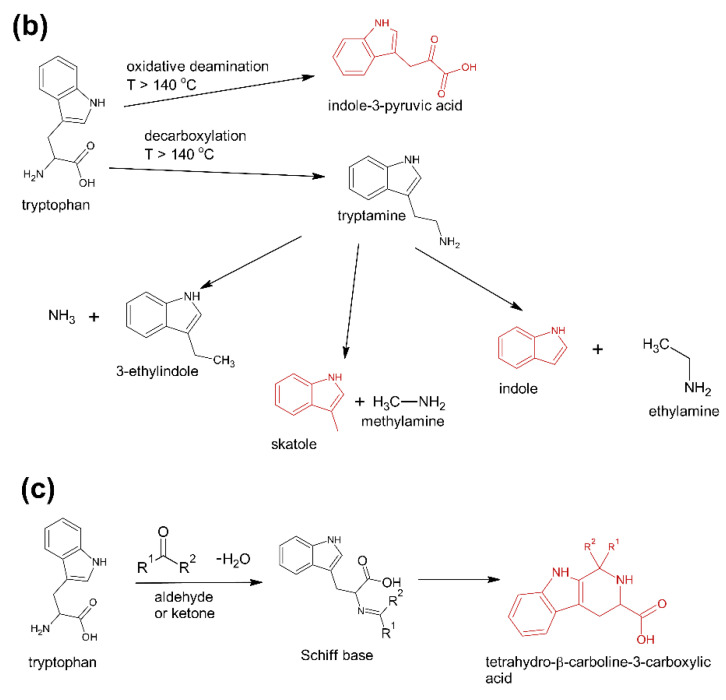
Pathways reasoning for the presence of specific contaminants in Trp supplements. (**a**) Major metabolic pathways downstream of Trp (**b**), Trp degradation products formed after exclusive exposure to heat (**c**), and reaction products of Trp with aldehydes and ketones. Compounds marked in red were detected in this study [37].

The first group (group A) includes products of the main metabolic pathways of Trp: anthranilic acid (**I4**), indole-3-acetaldehyde (**I7**), indole acetic acid (**I9**), 5-hydroxyTrp (**I16**), formylkynurenine (**I19**) (Figure 1).

These compounds are formed during the fermentation of Trp in biotechnological production through the activity of Trp-degrading enzymes. The second group (group B) of contaminants corresponds to the condensation of Trp with carbonyl compounds: tetrahydro-β-carboline-3-carboxylic acid (**I15**) (condensation with formaldehyde), 1-methyl-tetrahydro-β-carboline-3-carboxylic acid (**I17**) (condensation with acetaldehyde), 1-(3-methyleneindole)-tetrahydro-β-carboline-3-carboxylic acid (**I21**) (condensation with indole-3-acetaldehyde (**I7**)). Reactions of tryptophan with aldehydes/ketones to form tetrahydro-beta-carbolines (tetHβCs), known as the Pictet–Spengler reaction, is one of the most common reactions of tryptophan with organic compounds. The transformation is usually acid-catalysed and takes place at low pH and high temperatures [38]. Highly reactive aldehydes are generated during the fermentation processes, thus tetHβCs may be detected in any biotechnologically derived Trp [39]. In the summary, the first and second groups of contaminants are associated with the process of Trp production using fermentation. The third group (group C) contains indole (**I1**), skatole (**I2**), oxindole (**I3**), 3-formylindole (**I5**), unsaturated Trp (**I13**), indole pyruvic acid (**I14**), 2-(3-Methyleneindole)Trp (**I20**), 1-(2-Trp)-1-(3-indole)propanediol (**I22**), and originate from Trp degradation [37]. Indole (**I1**), skatole (**I2**), and indole pyruvic acid (**I14**) are Trp degradation products formed after exclusive exposure to heat. At temperatures above 140 °C, decarboxylation and oxidative deamination of Trp occurs, forming tryptamine and indole pyruvic acid (**I14**). Tryptamine can degrade further, to form a possible product: indole (**I1**) or skatole (**I2**) [39]. Additionally, contaminants from this group may be the precursors in the Trp production process as chemical synthesis (indole (**I1**), 3-formylindole (**I5**)), enzymatic synthesis (indole (**I1**)), biotechnological synthesis (indole (**I1**), anthranilic acid (**I4**)). The next group of contaminants (group D) is degradation products of kynurenine following irradiation and heat: 2,3-dihydro-4-quinolone (**I6**), kynurenic acid (**I11**), kynurenine yellow (**I12**). Thermal and UV radiation cause a cascade of reactions of kynurenine, and it transforms to yield kynurenine yellow (**I12**) and 4-quinolone. Kynurenine yellow (**I12**) can react further, undergoing either oxidative decarboxylation to also afford 4-quinolone or oxidation to kynurenic acid (**I11**) [40]. Compounds from the last group (group E) are probably accidental contaminants related to production conditions, packaging method or quality, transport conditions. The contaminants include 1-phenyl-3-methyl-5-pyrazolone (**I8**), glucosamine (**I10**), melatonin (**I18)**. The properties of some of them can be found in the literature. Glucosamine (**I10**) is used in the treatment of osteoarthritis [41]. Melatonin (**I18**) is centrally produced by the pineal gland and directly released in the blood, acting as a hormone. In mammals, yeast, and bacteria, melatonin **(I18**) is synthesized from tryptophan. Melatonin (**I18**) has a lot of functions: circadian and seasonal timing of organism; sleep and wakefulness cycle; endocrine functions, such as energy metabolism, glycaemic control, blood lipid profile and reproduction [42]. Glucosamine (**I10**) and melatonin (**I18**) are ingredients of many dietary supplements. Manufacturers of C8 and T3 (where we detected glucosamine (**I10**)) produce also dietary supplements containing glucosamine (**I10**). Thus, its presence (**I10**) in C8 and T3 may be the result of the insufficient purification (e.g., washing) before the manufacturing process. The same conclusion can be made in case of melatonin contaminant (**I18**).

**Figure 2:**
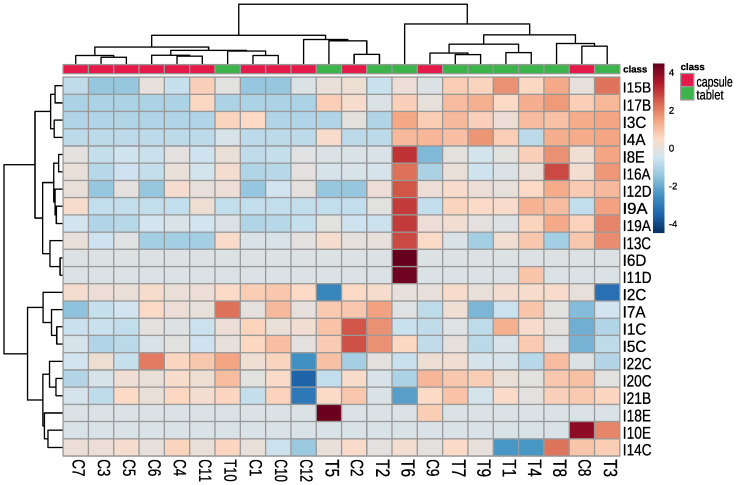
Clustering result of the tested supplements and detected contaminants (using Euclidean distance and clustering algorithm using Ward’s method). The level of contaminant is presented as a heatmap (red colour indicates higher concentration and blue colour indicates lower concentration than the average) T—tablet, C—capsule, I—contaminant (with the name of a group of contaminants, i.e., A—Trp’s metabolites, B—condensation products of Trp and carbonyls, C—Trp degradation products, D—degradation products of kynurenine, E—other contaminants).

To better visualize the results the heat map was prepared (Figure 2). We can observe the following:

a) Dietary supplements in capsules contained mainly contaminants from group C (Trp degradation products), which may indicate that Trp was obtained by chemical synthesis;
b) Dietary supplements in tablets contained mainly contaminants belonging to groups A (Trp’s metabolites) and B (condensation products of Trp and carbonyls), which may indicate that Trp was obtained by biotechnology;
c) Trp from C1 and C3 dietary supplements might be produced by the same manufacturer. The supplements contained the same contaminants (difference in **I3**—Trp degradation product, which may be related to different storage conditions);
d) Trp from C6 and C11 dietary supplements were produced by the same manufacturer, supplements contained the same contaminants (difference **I17**—condensation product of Trp and carbonyls);
e) Trp from C5 and C10 dietary supplements were produced by the same manufacturer, supplements contained similar contaminants, the differentiating contaminants were classified as Trp degradation products and can be generated during supplement storage.

To summarize, twenty-two compounds were detected in the analysed supplements in the range of 0.02% to 43.89% of the main ingredient area. Among the contaminants, there were Trp’s metabolites, condensation products of Trp and carbonyl compounds, Trp degradation products, degradation products of kynurenine, and other contaminants. Some of Trp’s contaminants have been already described in Trp raw material of different manufacturers [39], and melatonin supplements [1]. Melatonin can be synthesized from tryptophan by yeast and bacteria, so the occurrence of the contaminants was expected. The biological effect of Trp-related contaminants is unknown. Some Trp degradation products can impact cellular metabolism. **I12** a degradation product of Trp was shown to induce apoptosis in a human natural killer cell line. **I15** and **I17** act as antioxidants and free radical scavengers. However, the dose of **I12**, **I15**, **I17** needed to have a specific effect on cellular metabolism is unknown. The contaminants were present rather in small amounts, so they may not cause significant side effects [37].

Contamination can occur accidentally, due to poor manufacturing practices or contaminants originating from the supplement ingredients, or intentionally being added by manufacturers. The first group covers heavy metals [9] or substances found in raw materials, e.g., herbicides [43], insecticides [44], mycotoxins [45], and dioxins [11]. All detected contaminants in our study were from this group. Most of them were generated during manufacturing, under storage or transport of supplements/Trp, but some were found in the preparation by accident. Heavy metal analysis was not performed because it requires other analytical techniques such as ICP (inductively coupled plasma) or ASA (atomic absorption analysis). Moreover, these contaminants are mainly detected in herbal-based dietary supplements. Similarly, targeted screening for pesticides and mycotoxins (which were detected in herbal formulations), dioxins (detected in fish oil formulations), cyanobacterial neurotoxins (detected in shark cartilage) and microcystins (detected in algae) because they were not warranted, was not conducted in the study. For instance, pesticides were previously detected in supplements with Ginkgo [44,46] and Ginseng [47,48], whereas mycotoxins were in supplements with Ginkgo and grapes (Table A1). Many of these compounds require targeted screening as well as isolation and enrichment from the complex matrix to obtain a reliable signal [49]. The isolation methods include solid-phase extraction [50], dispersive solid-phase extraction [51], liquid-phase microextraction [52], microwave-assisted extraction [53], microwave-assisted saponification combined with simultaneous unsaponifiable extraction [54]. In our study, a simple extraction was performed without enrichment.

The second group of contaminants includes substances that are prohibited in dietary supplements and are intentionally used by the manufacturer to enhance the observed effect (anabolic steroids [10], hypoglycemic drugs [55], drugs used in potency disorders [56], weight loss products [57]). For Trp supplements, we screened for antidepressants because Trp is often used for depression [28], but no such substances were detected.

**Figure 3:**
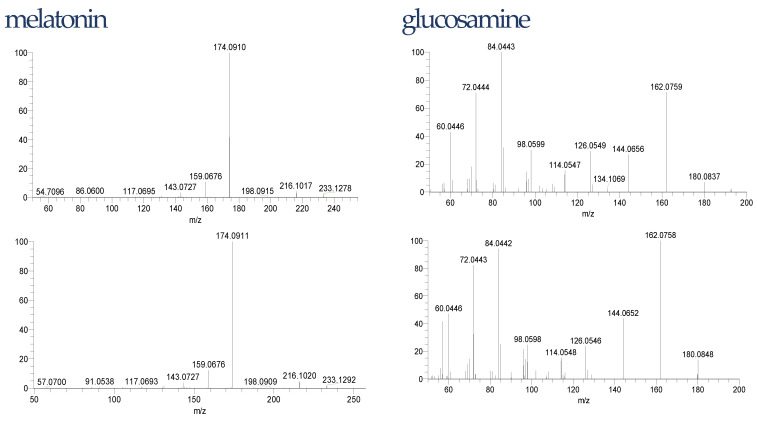
Glucosamine and melatonin fragmentation pattern in samples (**top**) and reference standards (**down**).

The liquid chromatography with mass spectrometry used in this experiment in the untargeted analysis is one of the most frequently used for that purpose. The increased bioavailability of high-resolution instruments improved the detection and identification of compounds in food including dietary supplements. However, confidence in these identifications varies between studies and substances, since it is not always possible or even meaningful to synthesize each substance or confirm them via complementary methods [58]. Thus, we applied the confidence identification level for our data (Table 1). To minimize the risk of false-positive identification it is recommended to search dedicated “small size” MS databases including compounds with a realistic probability to be observed [59]. In our case, the database consisted of degradation products of Trp was used. To decrease further the risk of false-positive identification, all detected compounds were fragmented to achieve a confidence level of at least 3. However, for unexpected compounds such as melatonin or glucosamine, we confirmed the structure with the reference standards. The differences in retention times of these compounds in the samples and reference standard were 0.01 min for glucosamine and 0.03 min for melatonin. The isotopic and fragmentation patterns were similar. The fragmentations are shown in Figure 3.

### 2.2. Determination of Trp in Dietary Supplements

Following the Polish Pharmacopoeia VI, the content of an active substance in tablets or capsules should not exceed the following: (1) ±10% for units with the declared active substance content below 100 mg or (2) ±5% for units with the declared content of the active substance of 100 mg and above. These requirements apply to pharmaceuticals. Due to the lack of specific guidelines for dietary supplements and the fact that dietary supplements appear in the same form as drugs, the same criteria for Trp content were adopted in this study. Therefore, none of the formulations contained the amount of Trp declared by the manufacturer (Table 2), i.e., it was not within 90–110% in each tablet or capsule. The lowest (55% of the declared content) Trp content was in supplement C6, followed by C8 (60%) and T5 (69%). The amount of Trp was within the range of 70–79% in nine supplements and 80–90% in the other nine supplements. The amount of Trp ranged from 70–79% in nine supplements and 80–90% in the other nine supplements. The low Trp content may be due to the lower amount of active ingredients used in production. The highest average Trp content (i.e., 100.45% of the claimed content) was observed in supplement C2. However, the amount of Trp in each capsule varied significantly and ranged from 174 to 251 mg/unit (CV = 19%), indicating improper mixing of the capsule mass. The concentration of the main ingredient in C6 and T2 supplements also had a high coefficient of variation: C6 (CV = 32%), T2 (CV = 35%), but the average amount of Trp was 55% and 87% of the claimed content, respectively. In these cases, both the wrong amount of active ingredient used and improper mixing of the tablet or capsule mass during the manufacturing process may be the reason for inadequate quality.

Inconsistency between the declared and determined content of the main ingredient has been previously reported for melatonin supplements [1] and lutein [8]. None of the lutein supplements (*n* = 10) and 41% of the melatonin supplements (*n* = 17) met our criteria. However, it is not clear whether the melatonin or lutein content was evenly distributed among the units. Therefore, no conclusions could be drawn regarding quality.

Content uniformity is an important critical quality attribute. High variability in active ingredient content can be caused by the following: improper particle distribution (e.g., agglomeration); poor macro- and microblending at the powder mixing stage; loss of a component (e.g., due to adsorption to the equipment surface); thief sampling and analytical errors; segregation of well-mixed blends during powder transfer, handling or further operations [60]. The controlling of all this process is required. In the case of pharmaceuticals available on the market, content uniformity is not likely to occur [61,62]. The only study showing the problem with this attribute concern tablets splitting [63]. However, a large number of articles on content uniformity and the ways of continuous monitoring tablet content uniformity [64] suggest that it is a difficult task to achieve.

For supplements with Trp, we observed only a slightly higher level of the active ingredient for capsules (79%) than for tablets (77%). Similar results were noted for melatonin (capsules—91%, tablets—87%) [1] and lutein (capsules—122%, tablets—42%). The supplements with lutein from Brazil (e.g., 0.12% or 135%) had lower quality than those from the USA (112%, 113%) [8].

### 2.3. Dissolution Test for Trp Tablets and Capsules

The Food and Drug Administration provides guidelines for drug testing. According to the dissolution test requirements, the active ingredient should release from the immediate-release oral solid drug at least 80% of its claimed content after 30 min of the release test [65].

**Figure 4:**
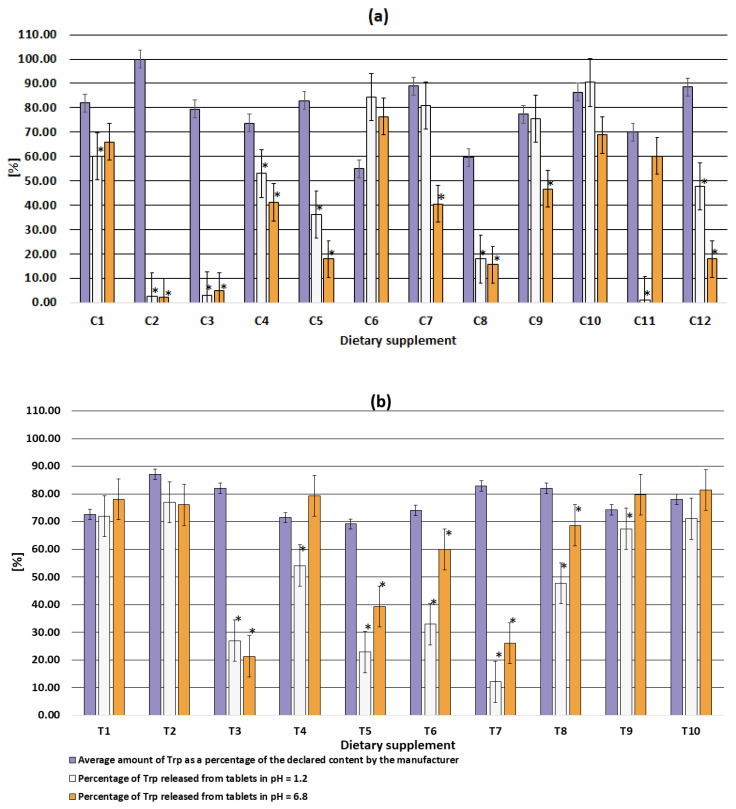
Comparison of the amount of Trp released at pH 1.2 (simulated gastric conditions), pH 6.8 (simulated intestinal conditions) with the amount determined in dietary supplements in capsules (**a**) and tablets (**b**); * significant differences (results not equal within the uncertainty) of the amount released with the amount detected.

Dietary supplements do not have dedicated guidelines for dissolution testing. Therefore, the same criteria for the dissolution test were used in this study. Trp release higher than 80% was determined for supplements C6, C7, C10 at pH 1.2 and supplement T9, T10 at pH 6.8. (Table 3, Figure 4). Supplement C11 had the lowest release (1.22%) at gastric pH (pH 1.2), while at pH 6.8 the release reached 60.2%. Supplements C2 and C3 were characterized by a release of no more than 5% Trp at both pHs (1.2 and 6.8). Thus, only up to 5% of the claimed dose of Trp could be absorbed from these supplements across biological membranes to produce a physiological effect (Figure 4a). Trp release between 10 and 20% regardless of dissolution medium was determined for C8 and between 20% and 30% for T3 and T7. In summary, Trp release for 10 of the 22 supplements was determined between 1.22 and 59.9% at both pHs (Figure 4).

Trp was completely released from C6, C10, T1, T2, T11 supplements at both pHs (Table 3, Figure 4). The other five supplements released Trp at only one pH: gastric (supplement C7, C9) or intestinal (supplement C1, C11, T4, T9). Thus, in these supplements, the amount of Trp released was limited only by the content of the main ingredient (Table 2). No negative effect of technological parameters and excipients was observed. One of these supplements, i.e., C11 was probably designed by the manufacturer as an enteral form, which was not even mentioned in the packaging. For this supplement, a release of less than 10% was observed at pH 1.2 (as recommended by the guidelines) and a complete release was observed at pH 6.8. However, due to the lower Trp content, the complete release did not reach 80% of the claimed content as recommended.

The low release of Trp from C2 (pH = 1.2, release 2.65%; pH = 6.8, release 2.3%) and C3 (pH = 1.2, release 3.08%; pH = 6.8, release 4.8%) was mainly due to improperly selected process parameters and/or improperly selected excipients. This is because the content of Trp in the dosage form was much higher than the amount of Trp released (Figure 4a). In the remaining formulations (i.e., C4, C6, C8, C9, C11, T1, T4, T5, T6, T9), the low Trp release was due to both low compound content in the formulation and inappropriate preparation technology (poorly selected technological parameters or excipients) (Figure 4). Referring to in vivo conditions, units characterized by low release will enter the gastric juice but will not release the substance. Thus, no physiological effect will be observed.

In summary, none of the analysed dietary supplements contained 80% or more Trp (for each tablet/capsule), which means that these formulations do not meet the release requirements for medicinal products. Comparative data on Trp release from other dietary supplements are not available. Applying our criteria to dietary supplements containing lutein [8], it also the case that none of these dietary supplements would meet these requirements. However, a comparison between the two studies is not easy because the release of the lutein supplements was performed using unconventional parameters. The dissolution test fluid for tablets was 2% P80 (*w*/*v*) and for capsules 2% P80 (*w*/*v*) with 25% ethanol. In the case of Trp supplements, the release test fluid was 0.1 mol/L hydrochloric acid (simulated gastric conditions) and 0.05 mol/L phosphate buffer (pH 6.8, simulated intestinal conditions) regardless of the form of the dietary supplement. Low release of active ingredients such as calcium carbonate [13], melatonin [14], folic acid [15,16], iron, zinc, manganese [17] and Grape seed extract [19], have also been observed in other dietary supplements in solid form (Table A2). However, in these cases, the reasons for the low release are not known due to the lack of data on the content of the main ingredient in these supplements. Low release may be due to insufficient content of the main ingredient, improperly selected process parameters, and/or improperly selected excipients. Only for food supplements containing triiodothyronine (*n* = 3) or prehormone thyroxine (*n* = 1) was the main component release above 93% [18].

In our study, Trp release was higher from tablets (12.1–81.4%) than from capsules (1.22–90.4%). Similar results were previously obtained for lutein [8] and folic acid [16]. For lutein supplements, release from capsules (made in the USA), despite containing adequate amounts of lutein, showed alarming results due to poor dissolution properties (less than 20% after 180 min of testing). These results may contribute to the lack of bioavailability of lutein. Unlike the capsules, the lutein tablets (made in Brazil) released more than 80% of the lutein within 180 min.

Analysis of the content, identity, and release of active ingredients from products is important to assess their quality [66]. The dissolution test determines the amount of active substance released and is mandatory for solid drug forms, but not for the same forms of dietary supplements [67]. The in vivo absorption of the active ingredient from solid formulations can be predicted to some extent using this assay [68]. A low release rate means low absorption and no intended effect. Thus, even the substance is in labelled amounts in the supplement but is not released, the consumer will not be able to achieve the effect.

Ease of marketing the supplement and low level of control combined with high popularity and high market value make dietary supplements a group of products particularly vulnerable to negligence or intentional manipulation, which poses a threat to consumers’ interests and sometimes even their health [21,69,70]. Determining the quality of dietary supplements is challenging and can be more difficult than for pharmaceutical products because such products often contain multiple vitamins, minerals [71], many of which are derived from plants [72] or other biological sources [73]. However, quality control of supplements should meet the same standards as pharmaceutical products because in both cases they are intended for consumer use [74]. The results of our studies developed with the use of a gold standard in analytics—mass spectrometry coupled with liquid chromatography, provide important data on the quality of the analysed dietary supplements. We hope that our results will encourage further research and increase public awareness about the purposefulness and safety of taking dietary supplements. An informed consumer will choose tested supplements, which will encourage manufacturers to test. In the case of the Food and Drug Administration, Good Manufacturing Practice in Manufacturing, Packing, Labelling, or Holding Operations for Dietary Supplements were already established. Applying GMPs to dietary supplements would be a further step to ensure products are consistently produced and controlled to the quality standards appropriate to their intended use [74].

A limitation of our study is the inability to detect compounds present at very low concentrations. These compounds require appropriate sample preparation. In addition, the targeted analysis should be chosen over non-targeted screening in their case.

## 3. Materials and Methods

### 3.1. Samples

The study was conducted on twenty-two Trp supplements, which is 10% of all dietary supplements with Trp in tablets or capsules registered in Poland, and all available on the market. There were two types of dosage forms: capsules (C1–C12) and tablets (T1–T10). All supplements were manufactured in the EU (Poland, UK, France, Germany, Czech Republic) and the USA. Six supplements were purchased in a Polish online e-commercial platform, the rest in pharmacies or online pharmacies in Poland.

### 3.2. Reagents

L-Trp (≥99%) (standard) and doxepin hydrochloride (internal standard) (≥98%) were purchased from Merck (Darmstadt, Germany). Hydrochloric acid (35–38%) solution pure p.a., sodium hydroxide (≥98.8%) pure p.a., potassium phosphate monobasic (≥99.5%) pure p.a. were purchased from Chempur (Piekary Śląskie, Poland). HPLC-grade methanol, acetonitrile, and formic acid were purchased from Merck (Darmstadt, Germany).

### 3.3. Sample Preparation

Three tablets or capsules were randomly selected from each supplement. The total weight of three tablets, or of the contents of three capsules, were determined. For tablets, a grinding step was applied. In the next step, the tablet’s mass or capsule content equivalent to 10 mg Trp was weighed and 1.00 mL of acetonitrile/methanol/water (1:1:1; *v*/*v*/*v*) mixture was added. The mixture was sonicated for 15 min and centrifuged for 5 min. The supernatant was then diluted with mobile phase to a concentration of 500 ng/mL or 100 ng/mL for qualitative and quantitative analysis, respectively. For quantitative analysis, an internal standard (doxepin) was added in the last step to a final concentration of 500 ng/mL.

### 3.4. Qualitative Analysis

Instrumental analysis was performed using a UHPLC Dionex Ultimate 3000 with a Q-Exactive hybrid quadrupole-orbitrap mass spectrometer system equipped with heat electrospray ionization (HESI), an online vacuum degasser, a quaternary pump, an autosampler, and a thermostatted column compartment. The HESI was operated in positive mode. Full MS scans were acquired over the *m*/*z* 100–1400 range with a resolution of 70,000 (*m*/*z* 200). Fragmentation was performed in different runs with a normalized collision energy of 20, 35, 50 eV. The ion selection threshold was 8 × 10^3^ counts, and the maximum allowed ion accumulation times were set to auto both for full MS scans and for the tandem mass spectrum. Standard mass spectrometric conditions for all experiments were: spray voltage, 3.5 kV; sheath gas pressure: 60 arb; aux gas pressure: 20 arb; sweep gas pressure: 0 arb, heated capillary temperature: 320 °C; loop count: 3; isolation window: *m*/*z* 1.0; and dynamic exclusion: 6.0 s. For all full scan measurements, lock-mass ions from ambient air (*m*/*z* 445.1200 and 291.2842) were used as internal calibrants.

Chromatographic separation was achieved with an Accucore C-18 column (100 mm × 4.6 mm, 2.6 µm) supplied by Thermo Fisher Scientific (Waltham, MA, USA) equipped with a security guard. The column was maintained at 40 °C at a flow rate of 0.3 mL/min. The mobile phases consisted of HPLC grade water with 0.1% formic acid as eluent A and acetonitrile with 0.1% formic acid as eluent B. The gradient (% B) was as follows: 0 min 10%; 1 min 10%; 10 min 95%; 15 min 95%. The volume of injection was 10 µL.

The results obtained were analysed using Compound Discoverer 3.0 software supplied by Thermo Fisher Scientific (Waltham, MA, USA).

The structures of the metabolites were proposed based on:

1. The *m*/*z* of the compound. The difference between experimental and theoretical molecular weight should be no higher than 5 ppm;
2. The isotopic pattern. The relative intensity tolerance to be used for the isotope search was set at 30%;
3. Fragmentation of the compound. The fragmentation spectrum was compared with experimental data found in the mass spectra library or the literature (confidence level 2), in silico fragmentation (confidence level 3) or reference standard (confidence level 1).

### 3.5. Quantitative Analysis

The instrumental analysis was performed using an Agilent 1260 Infinity (Agilent Technologies, Santa Clara, CA, USA), equipped with a degasser, autosampler, and binary pump coupled to a QTRAP 4000 hybrid triple quadrupole/linear ion trap mass spectrometer (AB Sciex, Framingham, MA, USA). The Turbo Ion Spray source was operated in positive mode. The curtain gas, ion source gas 1, ion source gas 2, and collision gas (all high-purity nitrogen) were set at 0.24 MPa, 0.41 MPa, 0.28 MPa, and “medium” instrument units, respectively. The ion spray voltage and source temperature were 4500 V and 600 °C, respectively. The target compounds were analysed in multiple reaction monitoring (MRM) mode. The compounds parameters, viz. declustering potential (DP), collision energy (CE), entrance potential (EP), and collision exit potential (CXP), were 76, 27, 12 V and 71, 25, 14 V for Trp and doxepin, respectively.

Chromatographic separation was achieved with a Kinetex C18 column (100 mm × 4.6 mm, 2.6 μm, Phenomenex, Milford, MA, USA). The injection volume was 10 μL. The flow rate was 0.75 mL/min. The mobile phases consisted of HPLC grade water with 0.2% formic acid as eluent A and acetonitrile with 0.2% formic acid as eluent B. The gradient (% B) was as follows: 0 min 5%; 1 min 5%; 2 min 95%; 3 min 95%.

The analysis of the Trp content in dietary supplements was preceded by method validation. The parameters tested were selectivity, precision, accuracy, linearity, and limit of quantification. The range of the calibration curve was selected as 0.01–10 µg/mL. Accuracy and precision were determined in triplicate at four concentration levels (0.01, 0.05, 5.0 and 10.0 µg/mL).

Calculations were made using the Analyst 1.6.3 software (AB Sciex, Framingham, MA, USA).

### 3.6. Dissolution Test for Tablets or Capsules

Trp release study was performed using a USP II Varian VK 7025 or USP I Varian VK 7025 dissolution tester (Erweka GmbH, Heusenstamm, Germany) for tablets and capsules, respectively. Six tablets or capsules were randomly selected and individually placed in the dissolution vessels. Each vessel contained 900 mL of dissolution medium. The stirring speed of 50 rpm or 100 rpm was used for tablets and capsules, respectively. The temperature was set at 37 ± 0.5 °C. Aliquots (1.5 mL) of the medium were manually collected using 5 mL syringes after 30 min of the test and filtered through a Millex-HA 0.45 µm filter. Each aliquot withdrawn was replaced with 1.5 mL of fresh medium. The experiment was performed both in hydrochloride acid pH 1.2 (simulated gastric conditions) and phosphate buffer pH 6.8 (simulated intestinal conditions). The Trp content was measured as described in Section 3.5 (Quantitative analysis).

### 3.7. Expanded Uncertainty

To assess whether the amount of Trp in the dosage unit and amount of the compound released is equal within the uncertainty range, extended uncertainty was determined using Equation (1).

(1) $$U\left(x_{1}-x_{2}\right)=2\;\sqrt{{\left[u(x_{1})\right]}^{2}+{\left[u(x_{2})\right]}^{2}}$$

The measurement results were equal if:

$$\left|x_{1}-x_{2}\right|<U\left(x_{1}-x_{2}\right)$$

$x_{1}$—mean [mg] Trp content determined in dosage unit using quantitative analysis (*n* = 3).

$x_{2}$—mean [mg] amount of Trp released from six dosage units.

$u\left(x_{2}\right),u\left(x_{1}\right)$—standard uncertainties of the measured values: $x_{1}$ and $x_{2}$ determined according to the formula:

$$u\left(x_{1}\right)=\frac{S}{\sqrt{n}}$$

S—standard deviation of the average amount of Trp in dosage unit [mg] or standard deviation of the released amount of Trp [mg].

*n*—the number of tablets or capsules analysed.

## 4. Conclusions

A new analytical approach based on liquid chromatography coupled to mass spectrometry provided the opportunity to obtain reliable results on the quality of dietary supplements. The quality of supplements is lower than that of pharmaceuticals with lower than claimed amounts of the main ingredient and a lack of uniform distribution between units. Sometimes, the release of the main ingredient is low, resulting in a lower probability of absorption and physiological effect. Contaminants were detected in all dietary supplements analysed, based on untargeted analysis. These substances, in the amounts determined, may not affect health or show significant unknown effects. The study confirms issues with the quality of dietary supplements and provides an important contribution to the discussion on the regulation of dietary supplements. We believe that the new analytical approach will have broad applicability in the assessment of supplement quality.

## Figures and Tables

**Table 1:** Tentative identification of contaminants found in Trp supplements and their MS parameters.

Code	Formula	Neutral Mass Calculated from the Formula [Da]	Neutral Mass Calculated from the Measured *m*/*z* [Da]	ΔMass [ppm]	RT [min]	Identification Confidence Level	Fragments [*m*/*z*]	Dietary Supplements Containing Contaminant (% of the Analysed)	Tentative Name	% of the Main Ingredient Area
**I1**	C8H7N	117.05785	117.05792	0.6	5.0	3	91.05414	All, (100%)	Indole	23.19
**I2**	C9H9N	131.07350	131.07350	0.0	5.0	2	117.06720; 130.06493; 131.07260	All (100%)	Skatole	6.68
**I3**	C8H7NO	133.05276	133.05243	2.5	3.1	2	79.05412; 106.06493	C1; C2; C8; C9; T1; T3; T4; T6; T7; T8; T9; T10 (55%)	Oxindole	2.41
**I4**	C7H7NO2	137.04768	137.04766	0.2	3.1	2	92.04936; 94.06511; 110.06001	C8; C9; T1; T3; T5; T6; T7; T8; T9; T10 (45%)	Anthranilic acid	0.42
**I5**	C9H7NO	145.05276	145.05284	0.6	5.0	2	91.05412; 117.05762; 118.06503	All (100%)	3-formylindole	43.89
**I6**	C9H9NO	147.06841	147.06827	1.0	3.9	3	120.04422; 130.03930; 130.06487	T6 (4.5%)	2,3-dihydro-4-quinolone	0.02
**I7**	C10H9NO	159.06841	159.06845	0.3	5.0	2	130.06490; 132.080610; 142.06616	All (100%)	Indole-3-acetaldehyde	2.50
**I8**	C10H10N2O	174.07931	174.07924	0.4	3.9	3	132.04401; 147.09129; 157.07565	C1, C2, C3, C4, C5, C6, C7, C8, C10, C11, C12, T1, T2, T3, T4, T5, T6, T7, T8, T9, T10 (95%)	1-Phenyl-3-methyl-5-pyrazolone	0.20
**I9**	C10H9NO2	175.06333	175.06326	0.4	3.9	2	130.06479; 146.05980; 158.05960	C7, C11, T1, T2, T3, T4, T6, T7, T8, T8 (45%)	Indole acetic acid	0.15
**I10**	C6H13NO5	179.07937	179.07938	0.1	3.1	1	127.03854; 144.06540; 145.04945	C8, T3 (9%)	Glucosamine	0.07
**I11**	C10H7NO3	189.04259	189.04239	1.1	8.5	2	162.05463; 172.03886; 173.04672	T4, T6 (9%)	Kynurenic acid	2.33
**I12**	C10H9NO3	191.05824	191.05811	0.7	4.0	3	150.05463; 164.07000; 174.05472	C4, C5, C7, C8, C9, C10, C11, C12, T1, T2, T3, T4, T6, T7, T8, T9, T10 (77%)	Kynurenine yellow	0.03
**I13**	C11H10N202	202.07423	202.07414	0.4	3.9	3	130.06480; 157.07570; 185.07106	C1, C2, C3, C5, C7, C8, C9, C10, C12, T1, T2, T3, T4, T5, T6, T7, T10 (77%)	Unsaturated Trp	0.38
**I14**	C11H9NO3	203.05824	203.05849	1.2	3.9	3	160.07555; 176.07080; 186.05499	C1, C2, C3, C4, C5, C6, C7, C8, C9, C10, C11, C12, T2, T3, T5, T6, T7, T8, T9, T10 (91%)	Indole pyruvic acid	0.03
**I15**	C12H12N2O2	216.08988	216.08986	0.1	8.0	3	171.09120; 173.10748; 188.07051	C2, C4, C6, C7, C8, C9, C11, C12, T1, T2, T3, T4, T5, T6, T7, T8, T9, T10 (82%)	Tetrahydro-β-carboline-3-carboxylic acid	0.23
**I16**	C11H12N2O3	220.08479	220.08467	0.5	3.9	2	130.06488; 158.05981; 175.08636	All (100%)	5-hydroxyTrp	0.18
**I17**	C13H14N2O2	230.10553	230.10533	0.9	8.2	3	168.08034; 188.07034; 214.08580	C2, C8, C9, C11, T1, T2, T3, T4, T5, T6, T7, T8, T9 (59%)	1-methyl-tetrahydro-β-carboline-3-carboxylic acid	0.02
**I18**	C13H16N2O2	232.12118	232.12099	0.8	9.1	1	174.09070; 204.10060; 216.10116	C9 (4.5%)	Melatonin	1.62
**I19**	C11H12N2O4	236.07971	236.07932	1.6	3.9	3	146.05975; 173.06980; 203.08099	All (100%)	n-formylkynurenine	0.49
**I20**	C20H19N3O2	333.14773	333.14739	1.0	8.8	3	188.07037; 205.09702; 217.09743	C1, C2, C3, C4, C5, C6, C7, C8, C9, C10, C11, T1, T2, T3, T4, T5, T6, T7, T8, T9, T10 (95%)	2-(3-Methyleneindole)Trp	0.02
**I21**	C21H19N3O2	345.14773	345.14745	0.8	9.0	3	283.12204; 285.13794; 329.12610	C1, C2, C3, C4, C5, C6, C7, C8, C9, C10, C11, T1, T2, T3, T4, T5, T6, T7, T8, T9, T10 (95%)	1-(3-methyleneindole)-tetrahydro-β-carboline -3-carboxylic acid	0.10
**I22**	C22H23N3O4	393.16886	393.16878	0.2	8.5	3	251.31799; 277.11810; 358.15448	C1, C2, C3, C4, C5, C6, C7, C8, C9, C10, C11, T1, T2, T3, T4, T5, T6, T7, T8, T9, T10 (95%)	1-(2-Trp)-1-(3-indole)propane diol	0.03

**Table 2:** Content of Trp in the dietary supplements (maximum error value above 40 was bolded).

Code	Dosage Form	Source	Declared Trp Content [mg/unit]	Determined Trp Content [mg/unit] ^a^	Maximum Error [%]
**C1**	capsule	United Kingdom	250	205 (CV = 5.0%)	−21
**C2 ^b^**	capsule	France	220	221 (CV = 19%)	−21
**C3**	capsule	United States	500	398 (CV = 4.1%)	−24
**C4**	capsule	Poland	500	368 (CV = 4.1%)	−29
**C5**	capsule	Poland	500	415 (CV = 13%)	−27
**C6**	capsule	No label	500	277 (CV = 32%)	**−74**
**C7**	capsule	Czech Republic	160	143 (CV = 16%)	−29
**C8**	capsule	Germany	50	29.8 (CV = 6.6%)	**−47**
**C9**	capsule	Poland	100	77.3 (CV = 8.4%)	−32
**C10**	capsule	United Kingdom	500	432 (CV = 5.4%)	−19
**C11**	capsule	United States	500	350 (CV = 14%)	**−44**
**C12**	capsule	United States	500	443 (CV = 8.7%)	−20
**T1**	tablet	Poland	100	72.6 (CV = 10%)	−32
**T2**	tablet	United States	1000	870 (CV = 35%)	**−48**
**T3**	tablet	Poland	40	32.6 (CV = 14%)	−27
**T4**	tablet	Poland	100	71.4 (CV = 7.0%)	−33
**T5**	tablet	Poland	167	115.5 (CV = 7.1%)	−36
**T6**	tablet	Poland	50	37.0 (CV = 9.0%)	−33
**T7**	tablet	Poland	50	41.4 (CV = 8.1%)	−24
**T8**	tablet	Poland	50	41.1 (CV = 16%)	−29
**T9**	tablet	Poland	50	37.1 (CV = 3.6%)	−29
**T10**	tablet	No label	200	155 (CV = 15%)	−35

CV—coefficient of variation; ^a^—mean (standard deviation *n* = 3); ^b^—three capsules were analysed, results (251 mg, 239 mg; 174 mg).

**Table 3:** Comparison of the amount of Trp determined and released from tablets and capsules in two pH (gastric, pH = 1.2 and intestinal, pH = 6.8) with the expanded uncertainty.

Code	The Average Percentage of Trp Amount Released from a Dosage Form (Standard Deviation *n* = 6)		Expanded Uncertainty Parameters					
			pH 1.2			pH 6.8		
	pH 1.2	pH 6.8	$\left|x_{1}-x_{2}\right|$	$U\left(x_{1}-x_{2}\right)$	Equal ^a^	$\left|x_{1}-x_{2}\right|$	$U\left(x_{1}-x_{2}\right)$	Equal ^a^
**C1**	60 (11)	66.0 (8.1)	55.38	25.29	No	39.99	136.07	Yes
**C2**	2.65 (0.55)	2.3 (2.2)	215.43	48.12	No	216.31	8.53	No
**C3**	3.08 (0.76)	4.8 (2.6)	382.17	19.07	No	373.66	57.22	No
**C4**	53 (13)	41.2 (3.8)	100.87	52.04	No	162.59	117.62	No
**C5**	36.2 (7.5)	17.9 (3.7)	234.17	68.74	No	325.37	113.25	No
**C6**	84.3 (8.3)	76.4 (2.2)	144.78	187.05	Yes	105.26	233.65	Yes
**C7**	81 (18)	40.6 (3.3)	13.18	37.53	Yes	77.73	9.87	No
**C8**	17.9 (1.8)	15.58 (0.70)	20.81	3.89	No	21.98	0.29	No
**C9**	75.5 (3.1)	46.6 (6.6)	1.75	10.06	Yes	30.65	15.13	No
**C10**	90.4 (9.4)	68.9 (7.3)	19.93	49.18	Yes	87.62	435.58	Yes
**C11**	1.22 (0.31)	60.2 (8.1)	344.27	80.11	No	49.64	541.99	Yes
**C12**	47.7 (5.8)	18.0 (1.5)	204.17	55.25	No	352.75	17.95	No
**T1**	71.9 (9.1)	78.0 (5.1)	0.7	11.42	Yes	5.41	9.64	Yes
**T2**	77.9 (5.4)	76.0 (5.7)	91.2	355.46	Yes	109.37	1071.86	Yes
**T3**	27 (16)	21.3 (6.9)	21.9	7.22	No	24.12	3.37	No
**T4**	54.1 (12.2)	79.3 (8.8)	17.3	11.55	No	7.93	26.61	Yes
**T5**	22.8 (2.9)	39.3 (6.2)	77.3	10.29	No	49.75	36.69	No
**T6**	32.9 (4.9)	59.9 (6.1)	20.5	4.31	No	7.01	3.94	No
**T7**	12.1 (1.0)	26.1 (2.4)	35.3	3.87	No	28.37	1.06	No
**T8**	47.7 (3.6)	68.3 (5.0)	17.3	7.84	No	6.80	2.85	No
**T9**	67.4 (7.1)	80.0 (5.9)	3.4	3.26	No	2.76	3.74	Yes
**T10**	71.0 (7.0)	81.4 (2.9)	13.3	28.43	Yes	7.49	11.42	Yes

^a^ amount of Trp in the formulation and amount of Trp released are equal (yes) or not (no) within the uncertainty.

## Data Availability

Data is contained within the article.

## Appendix A

**Table A1:** A review of research on contaminants in dietary supplements.

Type of Dietary Supplement	Number of Supplements	Contaminants	Country of Sale	Year	Method Applied	Ref.
**Plant based (e.g., Ginkgo biloba, Ginseng, flower pollen), algae**	24	Cd, Pb, Hg	Mexico	2007	ASA	[75]
**Mainly plant-based (herbs or botanicals as major components)**	95	As, Cd, Pb, Hg	USA	2003	ICP-MS	[76]
**Mainly plant-based (e.g., ginger, gingko biloba, ephedra), minerals**	40	Hg	USA	2005	ASA	[77]
**Plant-based and algae (e.g., gingko biloba)**	16	As	Denmark	2013	ICP-MS, LC-ICP-MS	[78]
**Iron supplements**	15	As	Brazil/Spain	2017	LC-ICP-MS	[79]
**Multimineral supplements**	168	Pb	Poland	2018	MIP-OES	[80]
**Herbal (improve hair, skin, and nails; regulate glucose levels)**	24	Hg	Poland	2018	ASA	[81]
**Prenatal and children supplements**	10	As	USA	2014	IC-ICP-MS	[82]
**Prenatal vitamin supplements**	51	As, Cd, Pb, Hg	Canada	2018	ICP-MS	[83]
**Health clays products**	27	As, Cd, Pb, Hg	Netherlands	2013	ICP-MS	[84]
**Calcium supplements**	45	Pb	USA	2007	ICP-MS	[85]
**Shark cartilage powder**	16	Cyanobacterial toxin (N-methylamino-L-alanine) and its isomers (2,4-diaminobutyric acid and N(2-aminoethy) glycine)), Hg	USA	2014	LC-FLD, LC–MS, CVAFS	[86]
**Ginkgo**	9	250 toxic substances including pesticides (e.g., hymexazol, tebufenozide) and mycotoxins (e.g., aflatoxin B1, aflatoxin B2, T-2 toxin), Insecticides, Fungicides, Herbicides	Spain, Poland, USA	2015	LC-HRMS	[46]
**Grape**	24	Mycotoxin (Ochratoxin A)	Italy	2015	LC–FLD	[87]
**Different plants (used for liver problems, menopause, for general health improvement)**	69	57 mycotoxins (e.g., zearalenone, enniatins)	Czech Republic, USA	2015	LC-MS	[88]
**Brewer’s yeast**	51	Mycotoxin (Ochratoxin A)	Germany	2002	LC-FLD	[89]
**Blue green algae**	17	Microcystins	Italy	2012	LC–MS, ELISA	[90]
**Blue green algae and Chlorella**	18	Microcystins	Germany	2012	PPIA, ELISA, LC–MS	[91]
**Ginseng**	23	Insecticide, Fungicides	USA	2016	GC-MS	[44]
**Soya**	14	Herbicides	Spain	2016	LC-MS	[92]
**Fish, seal and vegetable**	30	Insecticides	Canada	2009	GC-MS	[93]
**Omega-3**	9	Polychlorinated dibenzo-p-dioxins	Spain	2017	GC-MS	[94]
**Plant-based (weight loss)**	11	Sibutramine and its analogues, phenolphthalein	China	2008	LC-MS	[95]
**Plant-based (weight loss)**	24	Sibutramine and its analogues, rimonabant, phenolphthalein	Netherland	24	LC-DAD-MS	[96]
**Plant-based (naturally enhance sexual performance)**	74	PDE-5 inhibitors and their analogues	USA	2013	LC-DAD-MS	[97]
**Plant-based (enhance sexual potency)**	23	PDE-5 inhibitors and their analogues	Netherland	2013	LC-DAD-MS	[98]
**Tryptophan**	22	Untargeted screening, Trp products generated during production, storage, transport	Poland	2022	LC-HRMS	Current study

ASA—atomic absorption spectrometry, CVAFS—cold vapor atomic fluorescence spectrometry, DAD—diode array detection, ELISA—enzyme linked immuno-system, GC—gas chromatography, IC—ion chromatography, ICP-MS—inductively coupled plasma—mass spectrometry, LC-liquid chromatography, LC-HRMS—liquid chromatography–high resolution mass spectrometry, LC-FLD—liquid chromatography fluorescence detector, MIP-OES—microwave-induced plasma optical emission spectrometry, MS—mass spectrometry, PPIA—phosphatase inhibition assay.

**Table A2:** Review of studies on the release assay of the active substance from dietary supplements.

Main of Ingredient Dietary Supplement	Year	Country of Sale	Dosage Form	Number of Supplements	Dissolution Test	The Average Percentage of Trp Amount Released from a Dosage Form (Dissolution Medium)	Reference
**Calcium Carbonate**	1990	USA	tablet	27	Yes	5/27—below 75% (HCl pH 1.0) 4/27—between 33–75% (HCl pH 1.0) 18/27—less than 33% (HCl pH 1.0)	[13]
**Melatonin**	1999	USA	Immediate-release	9	Yes	4/9 above 75% (HCl pH 1.0)	[14]
			Controlled-release	2		½ above 90% (HCl pH 1.0)	
**Folic Acid**	2001	United Kingdom	capsule tablet	11	Yes	6/11—below 70% (0.1 M sodium hydroxide) 4/11—above 70% (0.1 M sodium hydroxide)	[15]
**Folic Acid**	2009	USA	tablet	14		45.0% (NaCl, pH 1.5) 104.5% (phosphate buffer, pH 7.5)	[16]
			capsule	1		15.2% (NaCl, pH 1.5) 47.4% (phosphate buffer, pH 7.5)	
**Iron, zinc, manganase**	2016	Poland	tablet	4	Yes	Iron—¼ above 80% (HCl, pH 1.2) Zinc—¼ above 80% (HCl, pH 1.2) Manganase—4/4–60% or less (HCl, pH 1.2)	[17]
**Lutein**	2018	Brazil USA	tablet	4	Yes	41.7% (2% polysorbate 80)	[8]
			capsule	6		122.5% (2% polysorbate 80 with 25% ethanol)	
**Triiodothyronine**	2019	United Kingdom	tablet	3	Yes	Above 93.5% (fasted-state simulated gastric fluid)	[18]
**Prehormone thyroxine**	2019	United Kingdom	tablet	1	Yes	Above 97.4% (fasted-state simulated gastric fluid)	[18]
**Grape seed extract**	2021	USA	capsule	1	Yes	73.09, 67.9, 71.06, 59.75% of gallic acid, catechin, procyanidin B2, and epicatechin, respectively (acetate buffer pH 4.6), 96.49, 89.09, 87.65, 78.84% of gallic acid, catechin, procyanidin B2, and epicatechinin, respectively (HCl pH 1.2)	[19]
**Trans-resveratrol**	2021	China	capsule	1	Yes	Above 75% (acetate buffer pH 4.6) Above 75% (HCl pH 1.2)	[19]
**Tryptophan**	2022	Poland	tablet	10	Yes	48.5% (HCl, pH 1.2) 61.0% (phosphate buffer pH 6.8)	Current study
			capsule	12		46.1% (HCl, pH 1.2) 38.2% (phosphate buffer pH 6.8)	
